# Supplementary material for: Engineering NIR-sighted bacteria
Source: eLife. 2025 Nov 3;14:RP107069. doi: 10.7554/eLife.107069 (PMC12582566; doi:10.7554/eLife.107069)
Supplement: Supplementary file 1. [file elife-107069-supp1.pdf]

Engineering NIR-Sighted Bacteria

Stefanie S. M. Meier[1,‡], Michael Hörzing[1,‡], Cornelia Böhm[2,3,‡], Emma L. R. Düthorn[1,‡], Heikki Takala[2,‡], René Uebe[4,5,‡], Andreas Möglich[1,5,6,*,‡]

[1] Department of Biochemistry, University of Bayreuth, 95447 Bayreuth, Germany.

[2] Department of Biological and Environmental Science, Nanoscience Center, University of Jyvaskyla, Jyvaskyla 40014, Finland.

[3] Institute of Biochemistry, Graz University of Technology, 8010 Graz, Austria

[4] Department of Microbiology, University of Bayreuth, 95447 Bayreuth, Germany.

[5] Bayreuth Center for Biochemistry & Molecular Biology, Universität Bayreuth, 95447 Bayreuth, Germany.

[6] North-Bavarian NMR Center, Universität Bayreuth, 95447 Bayreuth, Germany.

* for correspondence: andreas.moeglich@uni-bayreuth.de

‡ ORCID identifiers: S.S.M.M. 0009-0002-6028-5223; M.H. 0009-0008-2301-3783; C.B. 0000-0002-3552-2349; E.L.R.D. 0009-0000-4156-5428; H.T. 0000-0003-2518-8583; R.U. 0000-0003-2357-1589; A.M. 0000-0002-7382-2772

Contents

Supplementary Table A ____ 3

Supplementary Table B ____ 10

Supplementary Table A

Oligonucleotides used in this study. Overhang regions are indicated in bold, and restriction enzyme recognition sites are underlined.

Name	Sequence 5'-3'
Primers for cloning of bathy-PCM expression constructs	
M001_Ac_Bb_for	CCTGGTTGAAATTGTGCTGCGTCATAATGAGAATCTTTATTTTCAGCATCACCA
M002_Ac_Bb_rev	GATCAACTGCTTCTGCATTCGGCATGGTATATCTCCTTATTAAAGTTAAACAAAA
M003_Ac_Ins_for	TTTTGTTTAACTTTAATAAGGAGATATACCATGCCGAATGCAGAAGCAGTTGATC
M004_Ac_Ins_rev	TGGTGATGCTGAAAATAAAGATTCTCATTATGACGCAGCACAATTTCAACCAGG
M005_Av_Bb_for	ACCGTTAAAGTGGTTCTGCGCCATAATGAGAATCTTTATTTTCAGCATCACCA
M006_Av_Bb_rev	ATCAACGGTCTGGGTTGCAGGCATGGTATATCTCCTTATTAAAGTTAAACAAAA
M007_Av_Ins_for	TTTTGTTTAACTTTAATAAGGAGATATACCATGCCTGCAACCCAGACCGTTGAT
M008_Av_Ins_rev	TGGTGATGCTGAAAATAAAGATTCTCATTATGGCGCAGAACCACTTTAACGGT
M069_Pa-Ins-fw	TTTTGTTTAACTTTAATAAGGAGATATACCATGACGAGCATCACCCCGGTTACCC
M070_Pa-Ins-rev	TGGTGATGGCCCTGAAAATAAAGATTCTCCGCATGGTTGAGGCACAGTTCCATC
M071_Pa-Bb-fw	GATGGAACTGTGCCTCAACCATGCGGAGAATCTTTATTTTCAGGGCCATCACCA
M072_Pa-Bb-rev	GGGTAACCGGGGTGATGCTCGTCATGGTATATCTCCTTATTAAAGTTAAACAAAA
M017_AcAv_TEVrepair_fw	GGCCATCACCATCACCATCACCATCAC
M018_Ac_TEVrepair_rev	CTGAAAATAAAGATTCTCATTATGACGC
M019_Av_TEVrepair_rev	CTGAAAATAAAGATTCTCATTATGGCGC
Primers for Gibson cloning of bathy-PCMs into pREDusk	
M009_Ac_Bb_for	CCTGGTTGAAATTGTGCTGCGTCATAATCTCCAGGAACTGCAATCCGAGCTCGTC
M010_Ac_Bb_rev	TTGTCAGATCAACTGCTTCTGCATTCGTCATGCGTGGGCGACCTCAGGC
M011_Ac_Ins_for	GCCTGAGGTCGCCCACGCATGACGAATGCAGAAGCAGTTGATCTGACAA
M012_Ac_Ins_rev	GACGAGCTCGGATTGCAGTTCCTGGAGATTATGACGCAGCACAATTTCAACC

	AGG
M013_Av_Bb_for	CCGTTAAAGTGGTTCTGCGCCATAATCTCCAGGAACTGCAATCCGAGCTCGTC
M014_Av_Bb_rev	GTCAGATCAACGGTCTGGGTTGCAGTCATGCGTGGGCGACCTCAGGC
M015_Av_Ins_for	GCCTGAGGTCGCCCACGCATGACTGCAACCCAGACCGTTGATCTGAC
M016_Av_Ins_rev	GACGAGCTCGGATTGCAGTTCCTGGAGATTATGGCGCAGAACCACTTTAACG G
Primers for cloning of PATCHY *Ac*/*Av*NIRusk start constructs	
A07_P_I_II_Step1_for	TTAACCGAGCATCAGCAGACACAAGCACGTCTCCAGGAACTGCAATCCGAGC TCG
M020_AcREDusk_PATCHY_rev1	CGCTCGTCAGCCAGTAATTCATTATGACGCAGCACAATTTCAACC
M021_AcREDusk_PATCHY_rev2	GACGCACGTCTGCTTTCCCGCGCTCGTCAGCCAGTAATTCATTAT
M022_AcREDusk_PATCHY_fw2	AGCGTCTTCTTAATGAAGTTAACCGAGCATCAGCAGACACAAG
M023_AvREDusk_PATCHY_rev1	CGCTCCTCTTGCATCAGTTCATTATGGCGCAGAACCACTTTAACG
M025_AvREDusk_PATCHY_fw2	AGCGTGTCTTAAACGAGGTTAACCGAGCATCAGCAGACACAAG
M028_AvREDusk_PATCHY_rev2	GACGAATGTCTGCTTTGTTACGCTCCTCTTGCATCAGTTCATTAT
PATCHY forward primers for amplification of FixL	
A14_P_I_II_for_1	ACCGAGCATCAGCAGAC
A15_P_I_II_for_2	GAGCATCAGCAGACACAAG
A16_P_I_II_for_3	CATCAGCAGACACAAGC
A17_P_I_II_for_4	CAGCAGACACAAGCACG
A18_P_I_II_for_5	CAGACACAAGCACGTCTC
A19_P_I_II_for_6	ACACAAGCACGTCTCC
A20_P_I_II_for_7	CAAGCACGTCTCCAGG
A21_P_I_II_for_8	GCACGTCTCCAGGAAC
A22_P_I_II_for_9	CGTCTCCAGGAACTGC
A23_P_I_II_for_10	CTCCAGGAACTGCAATCC
A24_P_I_II_for_11	CAGGAACTGCAATCCG
A25_P_I_II_for_12	GAACTGCAATCCGAGC
A26_P_I_II_for_13	CTGCAATCCGAGCTCG
A27_P_I_II_for_14	CAATCCGAGCTCGTCC
A28_P_I_II_for_15	TCCGAGCTCGTCCACG

A29_P_I_II_for_16	GAGCTCGTCCACGTCTC
A30_P_I_II_for_17	CTCGTCCACGTCTCCAG
A31_P_I_II_for_18	GTCCACGTCTCCAGGC
A32_P_I_II_for_19	CACGTCTCCAGGCTGAG
A33_P_I_II_for_20	GTCTCCAGGCTGAGCG
A34_P_I_II_for_21	TCCAGGCTGAGCGCCATG
A35_P_I_II_for_22	AGGCTGAGCGCCATGG
A36_P_I_II_for_23	CTGAGCGCCATGGGCG
A37_P_I_II_for_24	AGCGCCATGGGCGAAATG
A38_P_I_II_for_25	GCCATGGGCGAAATGG
A39_P_I_II_for_26	ATGGGCGAAATGGCGTC
A40_P_I_II_for_27	GGCGAAATGGCGTCCG
A41_P_I_II_for_28	GAAATGGCGTCCGCGC
A42_P_I_II_for_29	ATGGCGTCCGCGCTCG
PATCHY forward primers for amplification of TtrS	
B92_P_Ttr_for_01	CGCGAACTGATTAATACCC
B93_P_Ttr_for_02	GAACTGATTAATACCCAGCG
B94_P_Ttr_for_03	CTGATTAATACCCAGCGTCAG
B95_P_Ttr_for_04	ATTAATACCCAGCGTCAGC
B96_P_Ttr_for_05	AATACCCAGCGTCAGC
B97_P_Ttr_for_06	ACCCAGCGTCAGCTGAATG
B98_P_Ttr_for_07	CAGCGTCAGCTGAATG
B99_P_Ttr_for_08	CGTCAGCTGAATGAAAATCG
C01_P_Ttr_for_09	CAGCTGAATGAAAATCGTG
C02_P_Ttr_for_10	CTGAATGAAAATCGTGCACTG
C03_P_Ttr_for_11	AATGAAAATCGTGCACTGC
C04_P_Ttr_for_12	GAAAATCGTGCACTGCTG
C05_P_Ttr_for_13	AATCGTGCACTGCTGG
C06_P_Ttr_for_14	CGTGCACTGCTGGAAC
C07_P_Ttr_for_15	GCACTGCTGGAACATGC
C08_P_Ttr_for_16	CTGCTGGAACATGCACAG
C09_P_Ttr_for_17	CTGGAACATGCACAGC
C10_P_Ttr_for_18	GAACATGCACAGCGTATTG
C11_P_Ttr_for_19	CATGCACAGCGTATTGC
C12_P_Ttr_for_20	GCACAGCGTATTGCCATTG

C13_P_Ttr_for_21	CAGCGTATTGCCATTGC
C14_P_Ttr_for_22	CGTATTGCCATTGCCG
C15_P_Ttr_for_23	ATTGCCATTGCCGGTG
C16_P_Ttr_for_24	GCCATTGCCGGTGAAC
C17_P_Ttr_for_25	ATTGCCGGTGAACTGG
C18_P_Ttr_for_26	GCCGGTGAACTGGGTG
C19_P_Ttr_for_27	GGTGAACTGGGTGCAAG
C20_P_Ttr_for_28	GAACTGGGTGCAAGCC
C21_P_Ttr_for_29	CTGGGTGCAAGCCTGAG
PATCHY forward primers for amplification of TodS	
C28_P_Tod_for_01	ACTGAGAAGAAACAAGCACAGG
C29_P_Tod_for_02	GAGAAGAAACAAGCACAGG
C30_P_Tod_for_03	AAGAAACAAGCACAGGAAAATC
C31_P_Tod_for_04	AAACAAGCACAGGAAAATCTTAACC
C32_P_Tod_for_05	CAAGCACAGGAAAATCTTAACC
C33_P_Tod_for_06	GCACAGGAAAATCTTAACCAG
C34_P_Tod_for_07	CAGGAAAATCTTAACCAGTTGC
C35_P_Tod_for_08	GAAAATCTTAACCAGTTGCAGC
C36_P_Tod_for_09	AATCTTAACCAGTTGCAGCAAC
C37_P_Tod_for_10	CTTAACCAGTTGCAGCAACAAC
C38_P_Tod_for_11	AACCAGTTGCAGCAACAAC
C39_P_Tod_for_12	CAGTTGCAGCAACAACTTG
C40_P_Tod_for_13	TTGCAGCAACAACTTGTG
C41_P_Tod_for_14	CAGCAACAACTTGTGTACG
C42_P_Tod_for_15	CAACAACTTGTGTACGTTTCC
C43_P_Tod_for_16	CAACTTGTGTACGTTTCCC
C44_P_Tod_for_17	CTTGTGTACGTTTCCCGATC
C45_P_Tod_for_18	GTGTACGTTTCCCGATCAG
C46_P_Tod_for_19	TACGTTTCCCGATCAGC
C47_P_Tod_for_20	GTTTCCCGATCAGCTACG
C48_P_Tod_for_21	TCCCGATCAGCTACGATG
C49_P_Tod_for_22	CGATCAGCTACGATGGG
C50_P_Tod_for_23	TCAGCTACGATGGGTGAATTTG
C51_P_Tod_for_24	GCTACGATGGGTGAATTTG
C52_P_Tod_for_25	ACGATGGGTGAATTTGC

C53_P_Tod_for_26	ATGGGTGAATTTGCAGC
C54_P_Tod_for_27	GGTGAATTTGCAGCCTATATTG
C55_P_Tod_for_28	GAATTTGCAGCCTATATTGC
C56_P_Tod_for_29	TTTGCAGCCTATATTGCACAC
PATCHY reverse primers for amplification of *Ac*PCM	
M029_Ac_rev1	TTCATTAAGAAGACGCTGACG
M030_Ac_rev2	ATTAAGAAGACGCTGACGC
M031_Ac_rev3	AAGAAGACGCTGACGC
M032_Ac_rev4	AAGACGCTGACGCACG
M033_Ac_rev5	ACGCTGACGCACGTCTG
M034_Ac_rev6	CTGACGCACGTCTGCTTTC
M035_Ac_rev7	ACGCACGTCTGCTTTCC
M036_Ac_rev8	CACGTCTGCTTTCCCG
M037_Ac_rev9	GTCTGCTTTCCCGCGC
M038_Ac_rev10	TGCTTTCCCGCGCTCG
M039_Ac_rev11	TTTCCCGCGCTCGTCAG
M040_Ac_rev12	CCCGCGCTCGTCAGCC
M041_Ac_rev13	GCGCTCGTCAGCCAGTAATTC
M042_Ac_rev14	CTCGTCAGCCAGTAATTCATTATG
M043_Ac_rev15	GTCAGCCAGTAATTCATTATGACG
M044_Ac_rev16	AGCCAGTAATTCATTATGACG
M045_Ac_rev17	CAGTAATTCATTATGACGCAGC
M046_Ac_rev18	TAATTCATTATGACGCAGCAC
M047_Ac_rev19	TTCATTATGACGCAGCAC
M048_Ac_rev20	ATTATGACGCAGCACAATTTC
PATCHY reverse primers for amplification of *Av*PCM	
M049_Av_rev1	CTCGTTTAAGACACGCTGAC
M050_Av_rev2	GTTTAAGACACGCTGACGAATG
M051_Av_rev3	TAAGACACGCTGACGAATG
M052_Av_rev4	GACACGCTGACGAATGTC
M053_Av_rev5	ACGCTGACGAATGTCTGC
M054_Av_rev6	CTGACGAATGTCTGCTTTG
M055_Av_rev7	ACGAATGTCTGCTTTGTTACG
M056_Av_rev8	AATGTCTGCTTTGTTACGC
M057_Av_rev9	GTCTGCTTTGTTACGCTCC

M058_Av_rev10	TGCTTTGTTACGCTCCTC
M059_Av_rev11	TTTGTTACGCTCCTCTTGC
M060_Av_rev12	GTTACGCTCCTCTTGCATC
M061_Av_rev13	ACGCTCCTCTTGCATCAG
M062_Av_rev14	CTCCTCTTGCATCAGTTCATTATG
M063_Av_rev15	CTCTTGCATCAGTTCATTATGG
M064_Av_rev16	TTGCATCAGTTCATTATGGC
M065_Av_rev17	CATCAGTTCATTATGGCGC
M066_Av_rev18	CAGTTCATTATGGCGCAG
M067_Av_rev19	TTCATTATGGCGCAGAACC
M068_Av_rev20	ATTATGGCGCAGAACC
Primers for cloning of orthogonal TtrSR TCS	
B55_Pttr_BglII_for	CGTTAAAGATCTATATTTGTTGCGCTAGA
B56_Pttr_XbaI_rev	TGCCAGTCTAGAACAGGCATTC
B57_Bb_for_DmTtrSR	ACGTCTGCGTCTGGAAGATAATCGTTAACAATTGATGTAAGTTAGCTCACTCA
B58_Bb_rev_DmTtrSR	CATTCAGCTGACGCTGGGTATTAATCAGCTCACGATTAGCACGCTCTAAG
B59_Ins_for_DmTtrSR	CTTAGAGCGTGCTAATCGTGAGCTGATTAATACCCAGCGTCAGCTGAATG
B60_Ins_rev_DmTtrSR	TGAGTGAGCTAACTTACATCAATTGTTAACGATTATCTTCCAGACGCAGACGT
B84_P_V_sc_for	GGCGTTTCGCCTTTGATATCGCGAACTGATTAATACCCAGCGTCAG
B85_P_V_sc_rev	GCAACTCTGCGTTACGCCCTTCAAGCTCACGATTAGCACGCTCTA
Primers for cloning of orthogonal TodST TCS	
TodX_fw	GCACAAGATCTGGTCTGAGGTTTTCATCGAC
TodX_rv	CGTGCTCTAGAAATTACAATCCTTCCAC
0_pDusk_fw	GAGTCAATTGAGGGTGGTGAATGTGGCTAG
1_TodS_fw	GATATCACCGAGAAGAAACAAGCACAGGAAAATC
2_NoMfeI_rv	CGTACACAAGTTGTTGCTGCAACTGGTTAAGATTTTCCTGTGCTTGTTTC
3_TodS_fw	GCAGCAACAACTTGTGTACGTTTCCCGATC
4_TodT_rv	GCATCAATTGCTATTCCAGGCTATCCTTGAG
5_pDusk_rv	GCTTGTTTCTTCTCGGTGATATCATTCTGAATTC
M014_Av_Bb_rev	GTCAGATCAACGGTCTGGGTTGCAGTCATGCGTGGGCGACCTCAGGC
M015_Av_Ins_for	GCCTGAGGTCGCCCACGCATGACTGCAACCCAGACCGTTGATCTGAC
C26_Bb_for_P_X_sc	ATTCGTCAGCGTGTCTTAAACGAGGTTAACTGAGAAGAAACAAGCACAG
C27_Ins_rev_P_X_sc	CTGTGCTTGTTTCTTCTCAGTTAACCTCGTTTAAGACACGCTGACGAAT

Primers for cloning StrR variants	
B73_Bb_for_pStrepDusk	GATCACCAAGGTAGTCGGCAAATAAGAATTAATTCATGAGCGGATACATATT
B74_Bb_rev_pStrepDusk	AAGGATCTCAAGAAGATCCTTTGATCTGATGCCTCCGTGTAAGGGG
B75_Ins_for_pStrepDusk	CCCCTTACACGGAGGCATCAGATCAAAGGATCTTCTTGAGATCCTT
B76_Ins_rev_pStrepDusk	AATATGTATCCGCTCATGAATTAATTCTTATTTGCCGACTACCTTGGTGATC
B26_Ins_rev	CGAGATGATAGGAGGTCTAGCATGACGACCAAGGGACATATCTACG
B27_Bb_for	CGTAGATATGTCCCTTGGTCGTCATGCTAGACCTCCTATCATCTCG
M014_Av_Bb_rev	GTCAGATCAACGGTCTGGGTTGCAGTCATGCGTGGGCGACCTCAGGC
M015_Av_Ins_for	GCCTGAGGTCGCCCACGCATGACTGCAACCCAGACCGTTGATCTGAC
Primers for cloning MCS variant	
C76_Bb_for_MCS	ATCAGCAACTACATGAAGGGCTCGCGGCGGCTGCTTGCCG
C77_Ins_rev_MCS	CGGCAAGCAGCCGCCGCGAGCCCTTCATGTAGTTGCTGAT
M014_Av_Bb_rev	GTCAGATCAACGGTCTGGGTTGCAGTCATGCGTGGGCGACCTCAGGC
M015_Av_Ins_for	GCCTGAGGTCGCCCACGCATGACTGCAACCCAGACCGTTGATCTGAC
Primers for cloning lux variants	
lux-op_Nde_for	**GGATCCCATATG** AAATTTGGAAACTTTTTGCTTACATACC
lux-op_Sac_rev	**TGACGAGCTC** TCAACTATCAAACGCTTCG
PfixK2_NdeI_for	**TGACCATATG** ATATCTCCTTCTTAAAGTTAAACAAAATT
PfixK2_rev_OLPmamG	**ACTAAGAGCTAGTAAAGCGAAAAAG** CGGGATCTCGACGCTCTC
DrHo_OLPmamG_for	**CTTTTTCGCTTTACTAGCTCTTAGTTCTCCAATAAATTCCCTGCGAAGCTTAGGAGATCAGTATATG** CTCCGACTCATGATCATGAAGC
FixJ_NotI_rev	**TGACGCGGCCGC** TCAATCGTTGAGCATGCC

Supplementary Table B

Plasmids used in this study.

Id	Construct	Reference
pBAM2	*p15A* ori, miniTn5, Amp^R, Kan^R	Uebe, unpublished
pBAM2-luxAE	*p15A* ori, miniTn5, Amp^R, Kan^R, *luxABCDE*	(Dziuba et al., 2021)
p*Dm*REDlux	*p15A* ori, miniTn5, Amp^R, Kan^R, PfixK2-*luxABCDE*, PmamDC-*DrhemO-Dmpcm-fixL*	(this study)
p*Av*NIRlux	*p15A* ori, miniTn5, Amp^R, Kan^R, PfixK2-*luxABCDE*, PmamDC-*DrhemO-Avpcm-fixL*	(this study)
pMH005	*Ac*NIRusk-0	(this study)
pMH006	*Av*NIRusk-0	(this study)
pMH008	pCDF-Duet-*Ac*PCM/HO	(this study)
pMH009	pCDF-Duet-*Av*PCM/HO	(this study)
pMH013	*Ac*NIRusk-PATCHY-start construct	(this study)
pMH014	*Av*NIRusk-PATCHY-start construct	(this study)
pMH017	*Ac*NIRusk+16a	(this study)
pMH018	*Ac*NIRusk+16b	(this study)
pMH019	*Ac*NIRusk-1a (*Ac*NIRusk)	(this study)
pMH020	*Av*NIRusk+16b	(this study)
pMH021	*Ac*NIRusk-4	(this study)
pMH023	*Av*NIRusk-1	(this study)
pMH026	pCDF-Duet-*Pa*PCM/HO	(this study)
pMH028	*Av*NIRusk+17a (*Av*NIRusk)	(this study)
pMH029	*Av*NIRusk+16a	(this study)
pMH030	*Av*NIRusk-5	(this study)
pMH034	*Ac*NIRusk-1b	(this study)
pMH038	*Av*NIRusk+17b	(this study)
pMIR66	TodS and TodT proteins	(Ramos-González et al., 2002)
pMIR77	PtodX promoter	(Ramos-González et al., 2002)

pQX037	*Pa*PAC	(Xu et al., 2024)
pQX086	*Av*PAC	(Xu et al., 2024)
pQX091	*Ac*PAC	(Xu et al., 2024)
pRD349	pDusk-YT1-X	(unpublished)
pSM001	*Dr*REDusk-MCS	(Multamäki et al., 2022)
pSM003	*Dm*REDusk	(Meier et al., 2024a)
pSM024	*Dm*REDusk-MCS	(Meier et al., 2024a)
pSM087	*Dm*DERusk	(Meier et al., 2024a)
pSM090	*Dm*DERusk-YPet	(Meier et al., 2024a)
pSM091	*Dm*DERusk-MCS	(Meier et al., 2024a)
pSM122	*Dm*DERusk-StrR-YPet	(this study)
pSM123	*Dm*Ttr-REDusk-PATCHY-start construct	(this study)
pSM128	*Av*Tod-NIRusk PATCHY-start construct	(this study)
pSM136	*Av*NIRusk-StrR-YPet	(this study)
pSM146	*Dm*Ttr-13	(this study)
pSM147	*Dm*Ttr-0	(this study)
pSM148	*Dm*Ttr+5	(this study)
pSM149	*Dm*Ttr+7a	(this study)
pSM150	*Dm*Ttr+7b	(this study)
pSM153	*Dm*Ttr+14	(this study)
pSM160	*Av*Tod+3	(this study)
pSM161	*Av*Tod+16	(this study)
pSM162	*Av*Tod+19	(this study)
pSM163	*Av*Tod+21	(this study)
pSM164	*Av*Tod+26	(this study)
pSM182	*Av*NIRusk-MCS	(this study)<br>Addgene: 235084
